# Perceived Psychological Impact on Children and Parents of Experiencing COVID-19 Infection in One or More Family Members

**DOI:** 10.3390/children9091370

**Published:** 2022-09-10

**Authors:** Paola Costenaro, Costanza Di Chiara, Valentina Boscolo, Alessia Barbieri, Alice Tomasello, Anna Cantarutti, Sandra Cozzani, Cecilia Liberati, Serenella Oletto, Carlo Giaquinto, Daniele Donà

**Affiliations:** 1 Division of Pediatric Infectious Diseases, Department for Women’s and Children’s Health, University of Padua, 35128 Padua, Italy; 2 Department of Statistics and Quantitative Methods, Division of Biostatistics, Epidemiology and Public Health, Laboratory of Healthcare Research and Pharmacoepidemiology, University of Milano-Bicocca, 20126 Milan, Italy

**Keywords:** COVID-19, children, adolescents, family cluster, SARS-CoV-2, resilience, psychological

## Abstract

SARS-CoV-2 infection may impair behavior and mental health; we explored the psychological impact on parents and children who experienced COVID-19 within their families. A cross-sectional web-based survey was conducted on families attending the COVID-19 Follow-up Clinic at the Department for Women’s and Children’s Health, Padua (Italy). From April 2020 to August 2021, 75 surveys were collected from 66 families (97 parents and 129 children); almost 70% of participants had COVID-19, mostly asymptomatic/mildly symptomatic, and the median time from infection to survey compilation was 164.7 days (SD 56). Most parents (>87%) reported positive relationships with family members either before, during, or after COVID-19. More than one-third of children and adolescents were unable to adapt to isolation. Among 31 pre-school children with a median age of 3 (SD 1.7), a change of one or more functions was reported for 74.2% of cases irrespective of COVID-19 status, particularly a change in circadian rhythm (25%), in relationship with parents (42.8%), and poor emotional control (36%). Among 74 children with a median age of 10.9 years (SD 2.7), 8.1% had a score indicating a disease; however, significant impairment in attention was reported for 16.7%, along with anxiety/depression and problems with conduct in 5.6% and 6.5% of cases, respectively.

## 1. Introduction

Coronavirus Disease 2019 (COVID-19), caused by the highly transmissible and pathogenic severe acute respiratory syndrome coronavirus 2 (SARS-CoV-2), has so far affected about 554 million people worldwide, leading to more than 6.3 million deaths (WHO Coronavirus [COVID-19] Dashboard, available at https://covid19.who.int/, accessed on 13 July 2022). The global spread of the COVID-19 pandemic has been severely affecting public health systems and economies worldwide, as well as changing our approach to infectious diseases.

Before the development of SARS-CoV-2 vaccines, the adoption of stringent social restrictions combined with other infection prevention measures were the only strategies to contain the viral spread, thus reducing SARS-CoV-2-related morbidity and mortality [1]. 

Such approaches slowed the spread of COVID-19, reducing the risk of healthcare resources becoming overwhelmed. On the other hand, social distancing mandates, such as lockdowns, led to a negative impact on the physical and mental health of individuals [2,3]. This sudden situation forced people to engage all their resources on an emotional-relational level to cope with the event [4]. Intra-family relationships are important to address the socio-relational changes and the health concerns felt, especially in the early stages of the pandemic [5]. 

Several studies reported high rates of stress, psychological distress, anxiety, and depression during the first year of the pandemic in both pediatric and adult populations, reflecting the impact of the COVID-19 pandemic on the mental health and well-being of children, adolescents, and adults [6,7,8,9,10]. Although preliminary evidence showed a correlation with immune dysfunction, including nonspecific neuroinflammation and antineuronal autoimmune dysregulation [11], recent studies observed no differences between infected and uninfected participants, highlighting that quarantine and social distance may play a role in the development of neurocognitive, behavioral, and mental health consequences, in the general population [10]. Firstly, it was shown that environmental changes, including reduction of spaces, lack of natural views, and reduction of sunlight exposure as experienced during quarantine, particularly in the first pandemic wave, had a relevant impact on people’s mental health [12,13]. In addition, the current COVID-19 pandemic has profoundly altered our lives in a very short period of time; the smart-working and homeschooling introduction, home-shopping implementation, and virtual meeting adoption lead to profound changes in lifestyle habits among individuals. Moreover, children and adults’ emotional well-being and mental health have been affected by several factors, such as the fear of unknown contagious diseases, anxiety for family members or friends infected with the novel SARS-CoV-2, limited access to health care services, and stigma of infectious diseases, leading to isolation from others and withdrawal [14].

Family daily routines have been strongly changed by the restrictive measures, including social isolation [15,16], resulting in changes in domestic dynamics [17]. The homeschooling conditions and the implementation of screen time and virtual meeting strategies among kids and adolescents have impacted caring skills, enhancing parental feelings of inability to care for their children [18]. In addition, parental worry related to losing their work, financial instability, and anxiety to return to a sense of normality could affect the partner’s relationship and consequently negatively impacted children’s mental health [19]. Moreover, since children and adolescents’ psychological well-being also depends on the educational and playful activities they usually carry out, the lockdown from COVID-19, with the interruption of daily experiences, reflected in behavioral consequences leading to social problems with peers and negative feelings [20,21]. Lastly, as previously described, the impact of COVID-19 as a severe disease among any family member may lead to an immediate or long-term negative impact on the other family members [22,23,24].

All these changes in daily life could impact the mental health of people, irrespective of whether they are affected by COVID-19 or not. Given the clinical and social relevance of family mental health, it becomes important to impart knowledge on the psycho-social impact of the current pandemic on adults and children, and to investigate the impact of social restrictions adopted during the pandemic on mental health. Furthermore, assessing the resilience of children to a stressful event would allow an understanding of the personal resources of children and adolescents to cope with difficulties. Herein, conducting an online survey on COVID-19 family clusters attending the Infectious Diseases Unit of the Department of Women’s and Children’s health of the University of Padua, we aimed to explore the psychological impact of the COVID-19 pandemic on Italian families. From this perspective, the primary aim of this study was to evaluate the psychological impact on children, adolescents, and their parents during the first year of the COVID-19 pandemic, evaluating their functional change and resilience to a stressful event never experienced before. The secondary aim of this study was to assess if behavioral changes differ among COVID-19-infected and uninfected children and older siblings.

## 2. Materials and Methods

### 2.1. Study Design and Participants

We provide findings from a single-center, cross-sectional study aimed at exploring the psychological impact of experiencing SARS-CoV-2 infection among one or more family members. The study was conducted on Italian families attending the COVID-19 Family Cluster Follow-up Clinic (CovFC) at the Department of Women’s and Children’s Health of the University Hospital of Padua (Veneto Region, Italy). From March 2020, families defined as “one or both parents with one or more children/older siblings sharing the same household” were referred to the Clinic by their family pediatrician (FP) 4-12 weeks after the end of isolation when meeting the following inclusion criteria of (a) having children of pediatric age, and (b) having at least one family member with a history of COVID-19. At enrolment, a pediatrician collected data on demographic parameters, past medical history, and vaccinal status, and performed a clinical evaluation. At each follow-up visit, blood samples were collected from all cases for serological assessment of SARS-CoV-2 infection, through either the detection of the anti-receptor binding domain (RBD) antibodies against SARS-CoV-2 spike protein (MAGLUMI™2000 Plus, Snibe Diagnostics, Snibe Diagnostics, New Industries Biomedical Engineering Co., Ltd [Snibe], Shenzhen, China.) and/or the quantification of SARS-CoV-2 neutralizing antibodies with a high throughput method for Plaque Reduction Neutralization Test (PRNT) [25].

**Figure 1:**
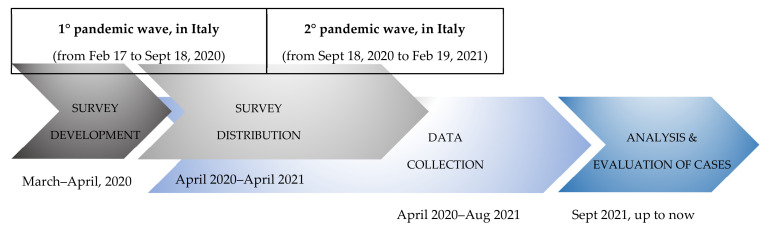
Study timeline, from survey development (from March to April 2020) to survey distribution (from April 2020 to April 2021) to the collection of the questionnaires after parent’s compilation (from April 2020 to August 2021) and analysis (from September 2021).

From March 2020 to April 2021, at the first evaluation, families were informed of their opportunity to be supported by a psycho-social team. Health Care Workers (HCWs) collected the consent of the parents/tutors of all families evaluated for suspected COVID-19 (among one/more family members) to be contacted to participate in the psycho-social project at the first clinical evaluation. After receiving their consent, a psychologist contacted each family by phone to explain the goal of the study; an ad-hoc questionnaire was further sent by email from April 2020 to April 2021 (Figure 1). Inclusion criteria for enrolling in the survey study were: (a) being enrolled at CovFC; (b) providing written consent for the use of the routine patient-based data for research purposes; (c) attendance of at least one follow-up visit; (d) at least one family member with confirmed COVID-19, at virological and/or serological assays. Exclusion criteria were (a) belonging to a family where all cases of suspected COVID-19 were not confirmed at further virological and/or serological assays (e.g., all members had negative COVID-19 results); (b) lack of written consent; (c) lack of attendance of at least one follow-up visit, at CovFC. The questionnaires were collected until August 2021 (Figure 1). The study was approved by the Ethical Committee of the Department (Prot. No. 0070714; amendment No. 71779). Parents or legally authorized representatives were informed of the research proposal and provided written consent to use the routine patient-based data for research purposes. 

### 2.2. Study Instruments

A web-based survey was developed and distributed online to a cohort of 88 COVID-19 family clusters enrolled at CovFC. The online survey was developed using the REDCap platform (Vanderbilt University, Nashville, TN, USA) hosted on the server of the University of Padua and was shared by email to all parents that explicitly agreed to participate in the study from April 2020 to April 2021 (Figure 1).

#### 2.2.1. Family Relationships Evaluation

A questionnaire was developed for retrospective data collection concerning the COVID-19 pandemic’s psycho-social impact on families and children’s resiliency. The survey included two main sessions. The first part, “Questionnaire A—Family”, was developed by a team including two psychologists, a social assistant, pediatricians, and an infectious diseases specialist. Parents were asked to describe the quality of the human relationship experienced within the family before, during, and after COVID-19, on a scale from 0 to 5 points (0 = worse, 1 = negative, 2 = more negative than positive, 3 = more positive than negative, 4 = positive, 5 = excellent).

#### 2.2.2. Child Emotional and Behavioral Problems and Adaptation to Home Isolation

“Questionnaire B/1–pre-school children” and “Questionnaire B/2—Pediatric Symptoms Checklist”. “Questionnaire B/1—pre-school children” was specifically developed by the team for children less than 6 years of age. Children’s ability to adapt to home isolation was investigated through a scale elaborated ad hoc, ranging from 0 to 5 (0 = very easy, 1 = quite well, 2 = easier than difficult, 3 = more difficult than easy, 4 = quite difficult, 5 = very difficult). For “Questionnaire B/2—Pediatric Symptoms Checklist”, the Pediatric Symptom Checklist (PSC) was used to evaluate children aged 6 to 17, to improve the recognition of psycho-social problems [21], as it was previously validated in the Italian population [26,27]. Thirty-five items parent-reported were proposed with a single choice answer of a score ranging from 0 to 1 (0 = never, 1 = sometimes, 2 = often). The PSC questionnaire provided total scores and three specific subscale scores: the “Attention Problems” subscale, which deepened attention and concentration impairment; the “Internalizing Problems” subscale, which investigated the anxiety and depressive symptoms; the “Externalizing Problems” subscale, which investigated the behavioral and conduct impairments. The “Attention Problems” subscale was derived from the sum of five specific items (“Fidgety, unable to sit still”, “Daydreams too much”, “Distracted easily”, “Has trouble concentrating”, “Hyperactive”) and a cut-off > 7 indicated an impairment condition. The “Internalizing problems” subscale was derived from the sum score of 4 items (“Feels sad or unhappy”, “Feels hopeless”, “Is down on him or herself”, “Worries a lot”), and a cut-off > 5 indicated an impairment condition. Lastly, the “Externalizing problems” subscale was derived from the sum of seven items (“fights with others”, “does not listen to rules”, “does not understand other people’s feelings”, “teases others”, “blames others for his/her troubles”, “takes things that do not belong to him/her”, “refuses to share”) and a cut-off > 7 indicated an impairment condition. The entire questionnaire is provided as Supplementary Materials.

All the questions were completed by a parent. For “Questionnaire A—Family”, both parents were allowed to provide their answers. However, for “Questionnaire B—Children”, specifically dedicated to children, only one parent was asked to provide answers. In the case of multiple children, the parent was asked to complete one survey for each child.

### 2.3. Clinical Data Collection and Definitions

Clinical data collected during follow-up visits were entered into a web-based database using the REDCap platform. Data were also collected retrospectively from the existing clinical files and analyzed anonymously. Participants were considered confirmed COVID-19 cases if they had a record of virological positivity for SARS-CoV-2 by real-time RT-PCR tests, and/or tested positive based on either of the two serological tests adopted in this study. For each confirmed COVID-19 case, a baseline date was defined as follows: (1) for symptomatic cases: the first date between the onset of symptoms or the date of first positive SARS-CoV-2 molecular assay; (2) for asymptomatic cases: the date of the first positive molecular assay or, in those with only serologically confirmed COVID-19 and with negative/undetermined nasal-pharyngeal (NP) swab, by the family outbreak temporal sequence, coinciding with the date of symptoms onset in the family cluster. Participants that were asymptomatic and had no analytical evidence of SARS-CoV-2 infection were considered non-COVID-19 cases. The severity of COVID-19 was scored as mild, moderate, severe, and critical, following the WHO classification [28].

Two periods of time or “COVID-19 waves” were identified and defined as follows: a first wave occurring from 17 February to 18 September 2020, and a second wave from 19 September 2020 to 18 February 2021 (Figure 1).

### 2.4. Statistical Analysis

A descriptive analysis of children and adults belonging to families that provided answers from at least one survey of the two proposed “Questionnaire A—Family” and “Questionnaire B—Children” was conducted. Counts and percentiles were provided for children and parents overall and stratified by survey respondents. Demographic data, comorbidities, COVID-19 diagnosis, clinical presentation, and time from dis-ease to survey compilation were evaluated.

Answers provided on “Questionnaire A—Family” were evaluated overall and stratified for COVID-19 pandemic waves (1st wave versus 2nd wave) according to the time of COVID-19 diagnosis, and the nonparametric Fisher exact test and the Mann-Whitney test were used to assess differences among either categorical or continuous covariates, respectively. To evaluate changes caused by the pandemic waves in the quality of family relationships, responses to the family relationships questionnaire on waves 1 and 2 were analyzed using nonparametric tests (Fisher’s exact test and Mann–Whitney test).

Data collected by the “Questionnaire B/1—pre-school children” and the “Questionnaire B/2—Pediatric Symptoms Checklist” were evaluated overall and stratified between confirmed COVID-19 versus COVID-19 negative cases. To evaluate differences in children’s emotional and behavioral health as a function of whether the child had a documented COVID-19 infection or not, scores on the “Questionnaire B/1—Pre-school children” and on the “Questionnaire B/2—Pediatric Symptoms Checklist” were compared using the Fisher exact test and the *t*-test. Statistical analysis was conducted using STATA 15.1, StataCorp, 4905 Lakeway Drive, College Station, TX USA; a *p* value <0.05 was considered statistically significant.

## 3. Results

**Figure 2:**
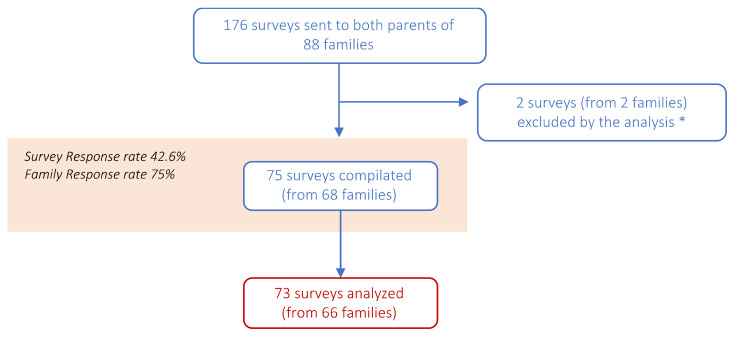
Flowchart describing the number of “Questionnaire A–Family” surveys distributed (*n* = 176) to 97 parents, and out of these, the number of surveys compilated (*n* = 75) and finally included in the analysis (*n* = 73). * Two surveys from 2 families were excluded by the analysis because: one family did not agree to be enrolled at CovFC and the other one attended the CovFC only once for probable COVID-19; however, both virological and serological assays resulted in negative for SARS-CoV-2 among all participants.

From April 2020 to April 2021, 176 web-based surveys were distributed by email to mothers and fathers of a cohort of 88 families attending the CovFC. Seventy-five surveys from 68 families were filled out, accounting for a 75% (68/88) family response rate: for 7 families, answers came from both parents (accounting for 14 surveys), while for the remaining 59 families, only one parent provided answers (accounting for 59 surveys). Two surveys from 2 families were excluded from the analysis (Figure 2). After submitting the questionnaire, 42 (47.7%) families required an online clinical psychological interview. In addition, for five children, the need for a psychological and/or psychiatric deepening was confirmed.

Overall, 66 families were included in the analysis, accounting for 97 parents with a median age of 43 years (SD 7.8) and 129 children with a median age of 10 years (SD 5.6). Among those, 74.2% of parents and 71.3% of children/adolescents had confirmed COVID-19, the majority presenting with mild symptomatic or asymptomatic infection. Seventy-three parents (66 families) answered “Questionnaire A–Family”. Of those, 58 (79.5%) were mothers, 15 were fathers with a median age of 43.3 years (SD 7.7), and 90.4% were Italian. Demographic characteristics of children and parents are reported in Table 1.

**Figure 3:**
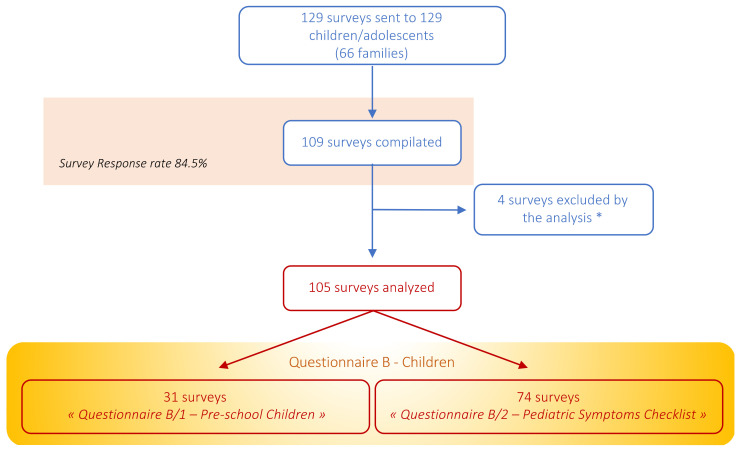
Flowchart describing the number of “Questionnaire B—Children” surveys distributed (*n* = 129), and out of these, the number of surveys compilated (*n* = 109) and included in the analysis (*n* = 105). * Four surveys were excluded from the analysis because they referred to adolescents ≥18 years of age.

Among the 129 Part B sent for completion, 109 surveys were completed, and 4 were excluded from the analysis as they referred to adolescents of ≥ 18 years of age (Figure 3).

### 3.1. Results from the “Questionnaire A—Family”

Findings from “Questionnaire A—Family” are reported in Table 2. Overall, the median time from COVID-19 disease (defined as the “baseline date”) to survey completion was 164.7 days (SD 56 days). Most families (95.8%) shared the diagnosis of COVID-19 with people other than close contacts. However, 53.4% of families were worried thinking about people’s behavioral changes after the communication of their diagnosis. In 30.5% of cases, people’s behavioral change towards the family was observed, with a significant negative impact for 72.7% of families that experienced COVID-19 during the 1st wave compared to those of the 2nd wave.

Overall, families reported a positive quality of relationships with partners, children, and siblings with no significant differences comparing the first and second pandemic waves. As expected, the most “critical” period was the time of COVID-19 infection, particularly during the 2nd wave, where 17.9% of parents experienced a negative relationship with their partner, 10.4% with children/adolescents, and 16% observed a negative relationship with siblings.

### 3.2. Results from the “Questionnaire B—Children”

Findings from the “Questionnaire B/1—Pre-school children” are reported in Table 3. Overall, 31 surveys were filled out by parents, referring to 31 children with a median age of 3 years (SD 1,7), 64.5% of those with confirmed COVID-19. Overall, a sub-optimal adaptation to isolation (≥3) was noticed for 35.5% of children, as indicated by their parents. During isolation at home, a change of one or more functions was reported for 74.2% of children, with no differences between COVID-19 positive and negative children. The most frequent alterations were a change in circadian rhythm (25%), a change in emotional expression and poor control (36%), and a change in relationship with both parents (42.8%) and siblings (28%). No differences were observed among COVID-19 positive and negative children.

Findings from the “Questionnaire B/2—Pediatric Symptoms Checklist” are reported in Table 4. Overall, among 82 children/adolescents aged ≥6 to <18 years, 74 surveys were filled out with at least one answer by their parents (90.2% response rate). The mean age of 74 children was 10.9 years (SD 2,7). Of those, 39.2% were female, and 22.9% had one or more comorbidities. Overall, a scarce ability to adapt to isolation (≥3) was noticed for 36.9% of children/adolescents, as indicated by their parents. For all cases, one or more functional changes were observed during isolation.

The Pediatric Symptom Checklist (PSC) was adopted based on previous studies [21,23]. Reaching a cut-off of ≥28 indicated a possible problem. In our cohort, 8.1% (6/74) of children were reported as having a score of ≥28, with no differences between confirmed COVID-19 cases and negative participants. The most common psycho-social alterations reported were “Tires easily, has little energy” (51.4%), “Less interested in school” (59.7%), “Distracted easily” (66.7%), “Is afraid of new situations” (51.4%), “Is irritable, angry” (54.2%), “Has trouble concentrating” (52.8%), and “Wants to be with you more than before” (54.3%). No differences among COVID-19 positive and negative cases were observed for all items.

Among all cases, 5 out of 69 (7.3%) children used to get hurt frequently, and all of them were COVID-19 cases. In addition, the inability to express emotions (“he/she does not show feelings”) was reported for 16 out of 69 (23,2%) children. Parents reported that 31.3% of children “blamed others for their troubles.

The scores relating to the “Attention Problems”, “Internalizing Problems”, and “Externalizing problems” subscales were then analyzed. On the “Attention Problems” subscale, reaching a cut-off > 7 indicates an impairment condition. In our cohort, Attention impairment was referred for 16.7% of the total. A trend (*p* = 0.06) was identified in the comparison between COVID-19 confirmed cases (18.3%) and negative (8.3%). On the “Internalizing problems” subscale, a cut-off > 5 indicates an impairment condition, and in our cohort, the anxiety and depressive impairment were referred for 4/72 (5.6%). On the “Externalizing problems” subscale (derived from the sum of “fights with others”, “does not listen to rules”, “does not understand other people’s feelings”, “teases others”, “blamed others for his/her troubles”, “takes things that do not belong to him/her”, “refuses to share”) a cut-off > 7 indicates an impairment condition. In our cohort, behavioral and conduct impairment was referred by parents for 4/62 of the total (6.5%). No difference between negative and confirmed COVID-19 cases was found, but all the internalizing and externalizing impairment conditions were referred for confirmed positive COVID-19 children and adolescents.

## 4. Discussion

Our study explored the psychological effects of the COVID-19 pandemic on Italian children and their parents. Using an electronically distributed survey, we evaluated the family’s relational and behavioral changes and the children’s ability to adapt to COVID-19 and home isolation among a cohort of 66 Italian family clusters of COVID-19 observed at the Department of Women’s and Children’s Health of the University Hospital of Padua during the first year of the SARS-CoV-2 pandemic.

We retrospectively investigated changes in children’s and parents’ behavioral well-being and daily routine according to parents’ perceptions. Despite more than half of parents being afraid of people’s behavioral changes after the communication of infection, almost all of them shared the COVID-19 diagnosis with others outside their family, both during the 1st and 2nd wave of the COVID-19 pandemic. However, more than half of the parents expressed concern about the possible change of attitude following this communication. In 30% of cases, they observed a change of attitude, particularly during the first pandemic wave. Compared to other infectious disease outbreaks that led communities to marginalize infected participants [29,30], our results showed that people would better assess disease-related stigma during the SARS-CoV-2 pandemic. This contrasts with other findings reporting a high level of social stigma among adults who recovered from COVID-19. Yuan et al. [31], in a cross-sectional study comprising 154 COVID-19 survivors and 194 healthy controls, observed that COVID-19-related stigma is still commonly experienced among COVID-19 survivors even though the outbreak has been well-contained. In this regard, it must be considered that our cohort was a self-selected sample of families who accepted the proposal to socialize and share their experience of the disease.

We further observed that all parents noticed at least one functional change in their child’s behavior during the lockdown. For children younger than 6 years, changes in emotional expression and control followed by a change in circadian rhythm were the most prominent, reported by approximately one-third and one-fourth of the parents, respectively. This data suggests that even preschoolers have been affected by the changes caused by isolation. In this developmental stage, the understanding of events passes through direct experience. For preschooler children, it could be difficult to construct a mental representation of the threat linked to the pandemic, as the infection is an invisible threat. Furthermore, for preschooler children, language might not represent the best tool for emotional expression. For these reasons, childhood alterations may appear nonspecific, and they may occur as alterations in body functions or the quality of emotional experiences.

According to the Pediatric Symptom Checklist (PSC) scores used in this study, an increase in lack of school interest, distractibility, tiredness and easy fatigue, anxiety, worry concerning a new situation, anger and irritability, and difficulty in concentrating were also noted in children older than 6 years and adolescents by approximately one third to one out of two of parents. For most school-age children and adolescents, parents stated a decreased interest in school activities, suggesting the primary importance of the school setting and the role of interpersonal and peer relationships in motivation to learn at any level of education. 

During lockdown restrictions, a change of one or more functions was reported by parents for almost 3 in 4 children under 6 years and all older children/adolescents. Approximately 1 in 6 youth had significant impairment in attention based on parent ratings. Our findings suggest that the children may have had a slow adaptation to the social changes suddenly imposed by the pandemic, as previously reported [5].

Overall, we found an 8.1% prevalence rate of psycho-social dysfunction as measured by the PSC in school-aged children, slightly lower than rates reported in previous studies [32,33] conducted in the general pediatric population and evaluated by the PSC parents’ version. Higher psychological distress scores were found in the youth self-report form PSC-17 in preadolescents and adolescents [24,34]. In our cohort, we can consider an underestimation effect linked to the younger age of our cohort (10 years of median age), the retrospective data collection, and the indirect collection through parents. 

We noticed no differences in resilience and functional changes in children and adolescents when they were stratified according to a COVID-19 diagnosis. This may reflect the significant role of the environmental restrictions adopted during the pandemic on children and adolescents’ behavioral changes rather than a disease-related impact.

However, we must underscore that almost all participants reporting anxiety-depressive and behavioral or conduct problems had a confirmed diagnosis of COVID-19, as for cases reporting attention problems. The lack of difference between COVID-19 infected and uninfected cases may be related to the small number of the uninfected group. We can also hypothesize that the COVID-19 diagnosis may have contributed to increasing the time of isolation both towards the outside world and within one’s own family, thus making the emotional and relational experience of participants with confirmed COVID-19 diagnosis more complex than those reported for negative cases. 

Despite being descriptive, our findings suggest that the severe quarantine measures adopted during the current pandemic may have a psychological impact on children and adolescents. These findings agree with other studies evaluating the psychological well-being consequences of the COVID-19 lockdown among Italian, Spanish and Chinese children [14,35,36]. 

Our study has several limitations. Firstly, a selection bias may have underestimated our results, as families were self-selected to participate in the survey. In fact, in our cohort, we did not observe families of children with significant disabilities and/or living with social-economic disadvantages. In contrast, almost all families observed belonged to the middle class and were very sensitive and motivated in attending the clinical follow-up. A second limitation concerns the fact that at the time of the survey’s distribution, all family members were healthy and well recovered from COVID-19; consequently, parents were retrospectively referring to the time of COVID-19, having time to rework the experience. Third, the survey was fully completed by parents; indeed, the results could reflect their perception of children and adolescents’ behavioral changes. In addition, while the PSC is a validated score used to evaluate mental health in children and adolescents, Questionnaire B1 was constructed ad hoc by our specialists; therefore, it needs to be validated for further studies. Finally, the limited sample of uninfected participants, compared to the COVID-19 infected groups, could have had a negative impact on detecting statistically significant differences. Thus, it should be noted that, unfortunately, this limitation has also undermined the power of this study to detect any clinically significant differences.

## 5. Conclusions

In conclusion, in our Italian cohort of COVID-19 family clusters, we observed the maintenance of satisfactory intra-family balance and relationships among households during a psychologically stressful event such as the lockdown. Moreover, we did not find any functional changes and mental health distress in children or adolescents, reflecting a good resilience in stressful situations among the pediatric population. 

Future studies, including a higher number of infected and uninfected children and adolescents, are needed to better evaluate the role of environmental changes, social distances, and severe lockdown measures on the mental health of the pediatric population. In addition, further studies evaluating the children’s and adolescents’ self-filled surveys are required to better understand their behavioral changes.

## Figures and Tables

**Table 1:** Demographic characteristics of 66 families (with overall 97 parents and 129 children/older siblings), including the 73 parents that completed the surveys “Questionnaire A–Family” and “Questionnaire B—Children”, defined as “survey’s editors”.

	Children (*n* = 129)	Parents	
		Overall (*n* = 97)	Survey’s Respondents (*n* = 73)
Time (days) from baseline to survey completion (mean, DS) *	-	-	164.71 (56.03)
**Age at survey completion (mean, DS)**	10 (5.6)	43 (7.8)	43.3 (7.2)
**Gender (female), n (%)**	58 (44.9%)	63 (64.9%)	58 (79.5%)
**Nationality**			
**Italian**	120 (93%)	89 (91.7%)	66 (90.4%)
**Other**	9 (6.9%)	8 (8.2%)	7 (9.6%)
**Comorbidities^§^**	22 (17%)	10 (10.3%)	10 (13.7%)
**Positive SARS-CoV-2 Nasal-Pharyngeal Swab (n, %)**	77 (59.7%)	64 (65.9%)	52 (71.2%)
**Confirmed COVID-19 (n, %)**	92 (71.3%)	72 (74.2%)	55 (75.3%)
**COVID-19 clinical presentation (among confirmed cases):**			
**asymptomatic**	20 (21.7%)	5 (6.9%)	4 (7.3%)
**mild COVID-19**	71 (77.2%)	61 (84.7%)	49 (89.1%)
**moderate/severe COVID-19**	0	4 (4.1%)	1 (1.8%)
**critical COVID-19**	1 (MIS-C)	1 (1%)	1 (1.8%)
**Pandemic wave**			
**1st wave**	72 (55.8%)	67 (69%)	44 (60.3%)
**2nd wave**	57 (44.2%)	30 (30.9%)	29 (39.7%)

* For parents who completed the survey with confirmed COVID-19, we evaluated the period occurred from “date of baseline” to “date of survey compilation”; in the cases where parents that were negative for COVID-19, we evaluated the period from the most recent “date of baseline” reported for a positive household to the date of survey compilation. ^§^ Children’s comorbidities were growth disorders (*n* = 2), prematurity (*n* = 2), asthma (*n* = 2), chronic lung disease (*n* = 1), congenital heart disease (*n* = 3), rheumatic disease (*n* = 1), or other [10]; none had congenital, acquired immune disease, or tumors.

**Table 2:** Results from the “Questionnaire A—Family” evaluating the quality of the human relationship experienced within the family. Results were provided by 73 parents from 66 families.

	Total (*n* = 73)	1st Wave (*n* = 44)	2nd Wave (*n* = 29)	*p*-Value *
Time from baseline to survey compilation, days (mean, DS)	164.7 (56)	165,3 (64.8)	163,7 (40.2)	0.1724
Diagnosis of COVID-19 was shared with people other than close contacts (yes/total answer)	69/72 (95.8%)	42/43 (97.7%)	27/29 (93.1%)	0.561
Fear of a people’s behavioural change, after the communication of COVID-19 (yes/total answer)	39/73 (53.4%)	22/44 (50%)	17/29 (58.6%)	0.485
Objective finding of people’s behavioural change, after the communication of COVID-19 (yes/total answer)	22/72 (30.5%)	11/43 (25.6%)	11/29 (37.9%)	0.304
Type of observed behavioral change (negative)	8/22 (36.4%)	8/11 (72.7%)	0/11 (0%)	0.001
**Before COVID-19**				
Quality of the relationship with the partner (≥3)	65/67 (97%)	38/39 (97.4%)	27/28 (96.4%)	1.000
Quality of the relationship with son/daughters (≥3)	73/73 (100%)	44/44 (100%)	29/29 (100%)	-
Quality of the relationship within siblings (≥3)	42/42 (100%)	17/17 (100%)	25/25 (100%)	-
**During COVID-19**				
Quality of the relationship with the partner (≥3)	59/66 (89.4%)	36/38 (94.7%)	23/28 (82.1%)	0.125
Quality of the relationship with son/daughters (≥3)	68/71 (95.8%)	42/42 (100%)	26/29 (89.6%)	0.064
Quality of the relationship within siblings (≥3)	34/39 (87.2%)	13/14 (92.9%)	21/25 (84%)	0.636
**At survey compilation**				
Quality of the relationship with the partner (≥3)	65/68 (95.6%)	38/40 (95%)	27/28 (96.4%)	1.000
Quality of the relationship with son/daughters (≥3)	72/73 (98.6%)	43/44 (97.7%)	29/29 (100%)	1.000
Quality of the relationship within siblings (≥3)	40/41 (97.6%)	15/16 (93.7%)	25/25 (100%)	0.390
**Responder was working before COVID-19 (yes)**	56/72 (77.8%)	36/44 (81.8%)	20/28 (71.4%)	0.386
Impact of COVID-19 on work (yes)	20/66 (30.3%)	13/39 (33.3%)	7/27 (25.9%)	0.593
Negative impact	8/9 ^§^			

* *p*-value for the Fisher exact test (for categorical variables) or Mann-Whitney test (for quantitative variables). ^§^ we specify that of those, only 1 person resigned from work. All percent values have been calculated according to the total answers (handling missing values).

**Table 3:** Findings from the “Questionnaire B/1—pre-school children” evaluate the children’s ability to adapt to home isolation and the functional changes that occurred during isolation. Results refer to 31 children less than 6 years of age.

	TOT (*n* = 31)	COVID-19 CASE (*n* = 20)	COVID-19 Negative (*n* = 11)	*p*-Value *
**Age at survey compilation, mean (DS)**	3.01 (1.71)	2.85 (1.86)	3.33 (1.43)	0.232
**Gender (female)**	14/31 (45.2%)	9/20 (45%)	5/11 (45.5%)	1.000
**Comorbidities (yes)**	5/31 (16.1%)	4/20 (20%)	1/11 (9%)	0.631
**Scarce ability to adapt to isolation (** **≥3)**	11/31 (35.5%)	7/20 (35%)	4/11 (36.4%)	1.000
**Functional change occurred during isolation (yes)**	23/31 (74.2%)	13/20 (65%)	10/11 (90.9%)	0.203
**Change in circadian rhythm (yes)**	7/28 (25%)	4/18 (22.2%)	3/10 (30%)	0.674
**Change in nutrition (yes)**	3/25 (12%)	3/15 (20%)	0/10 (0%)	0.250
**Change in sphincteric control (yes)**	2/22 (9.1%)	2/12 (16.7%)	0/10 (0%)	0.481
**Speech alteration (yes)**	3/26 (11.5%)	3/16 (18.7%)	0/10 (0%)	0.262
**Play alteration (yes)**	5/27 (18.5%)	4/17 (23.5%)	1/10 (10%)	0.621
**Change in body care (yes)**	4/23 (17.4%)	0/12 (0%)	4/11 (36.4%)	0.037
**Change in emotional expression and control (yes)**	9/25 (36%)	4/14 (28.6%)	5/11 (45.5%)	0.434
**Change in relationship with parents (yes)**	12/28 (42.8%)	6/17 (21.4%)	6/11 (21.4%)	0.441
**Change in relationship with siblings (yes)**	7/25 (28%)	4/14 (28.6%)	3/11 (27.3%)	1.000

* Fisher exact test for categorical variables, *t*-test for quantitative variables.

**Table 4:** Findings from the Pediatric Symptoms Checklist named “Questionnaire B/2—Pediatric Symptoms Checklist” refer to 74 children/adolescents aged ≥6 to <18 years.

	TOT (*n* = 74)	COVID-19 CASES (*n* = 62)	COVID-19 Negative (*n* = 12)	*p*-Value *
**Age at survey compilation, mean (DS)**	10.9 (2.7)	10.3 (2.7)	10.9 (2.7)	0.775
**Gender (female)**	29 (39.2%)	23 (37%)	6 (50%)	0.521
**Comorbidities (yes)**	17 (22.9%)	14 (22.6%)	3 (25%)	1.000
**Scarce ability to adapt to isolation (** **≥** **3)**	27/73 (36.9%)	23/61 (37.7%)	4/12 (33.3%)	1.000
**Functional change occurred during isolation, any (yes)**	74 (100%)	62 (100%)	12 (100%)	-
**Pediatric Symptom Checklist (PSC) scores of** **≥** **28**	6/74 (8.1%)	5/62 (8%)	1/12 (8.3%)	1.000
**Attention Problems subscale (** **≥** **7)**	12/72 (16.7%)	11/60 (18.3%)	1/12 (8.3%)	0.676
**Internalizing Problems subscale (** **≥** **5)**	4/74 (5.4%)	4/62 (6.5%)	0/12	1.000
**Externalizing Problems subscale (** **≥** **7)**	4/72 (5.6%)	4/60 (6.7%)	0/12	1.000
**Complains of aches/pains (** **≥** **1)**	23/70 (32.9%)	21/59 (35.6%)	2/11 (18.2%)	0.318
**Spends more time alone (** **≥** **1)**	28/71 (39.4%)	24/60 (40%)	4/11 (36.4%)	1.000
**Tires easily, has little energy (** **≥** **1)**	37/72 (51.4%)	33/60 (55%)	4/12 (33.3%)	0.214
**Fidgety, unable to sit still (** **≥** **1)**	34/72 (47.2%)	30/60 (50%)	4/12 (33.3%)	0.354
**Has trouble with a teacher (** **≥** **1)**	17/72 (23.6%)	15/60 (25%)	2/12 (16.7%)	0.719
**Less interested in school (** **≥** **1)**	43/72 (59.7%)	36/60 (60%)	7/12 (58.3%)	1.000
**Acts as if driven by a motor (** **≥** **1)**	24/72 (33.3%)	21/60 (35%)	3/12 (25%)	*0.739*
**Daydreams too much (** **≥** **1)**	31/72 (43.1%)	25/60 (41.7%)	6/12 (50%)	0.751
**Distracted easily (** **≥** **1)**	48/72 (66.7%)	40/60 (66.7%)	8/12 (66.7%)	1.000
**Is afraid of new situations (** **≥** **1)**	37/72 (51.4%)	31/60 (51.7%)	6/12 (50%)	1.000
**Feels sad, unhappy (** **≥** **1)**	26/72 (36.1%)	20/60 (33.3%)	6/12 (50%)	0.330
**Is irritable, angry (** **≥** **1)**	39/72 (54.2%)	33/60 (55%)	6/12 (50%)	0.762
**Feels hopeless (** **≥** **1)**	7/72 (9.7%)	6/60 (10%)	1/12 (8.3%)	1.000
**Has trouble concentrating (** **≥** **1)**	38/72 (52.8%)	32/60 (53.3%)	6/12 (50%)	1.000
**Less interest in friends (** **≥** **1)**	18/72 (25%)	15/60 (25%)	3/12 (25%)	1.000
**Fights with others (** **≥** **1)**	31/72 (43%)	29/60 (48.3%)	2/12 (16.7%)	*0.057*
**Absent from online school (** **≥** **1)**	16/72 (22.2%)	14/60 (23.3%)	2/12 (16.7%)	1.000
**School grades dropping (** **≥** **1)**	25/72 (34.7%)	22/60 (36.7%)	3/12 (25%)	0.524
**Is down on him or herself (** **≥** **1)**	16/72 (22.2%)	13/60 (21.7%)	3/12 (25%)	0.722
**Visits doctor with doctor finding nothing wrong (** **≥** **1)**	5/72 (6.9%)	3/60 (4.2%)	2/12 (2.8%)	0.191
**Has trouble sleeping (** **≥** **1)**	21/71 (29.6%)	17/59 (28.8%)	4/12 (33.3%)	0.740
**Worries a lot (** **≥** **1)**	36/74 (48.6%)	30/62 (48.4%)	6/12 (50%)	1.000
**Wants to be with you more than before (** **≥** **1)**	38/70 (54.3%)	32/59 (54.3%)	6/11 (54.5%)	1.000
**Feels he or she is bad (** **≥** **1)**	8/70 (11.4)	7/59 (11.9%)	1/11 (9.1%)	1.000
**Takes unnecessary risks (** **≥** **1)**	5/69 (7.3%)	5/58 (8.6%)	0/11	0.585
**Gets hurt frequently (** **≥** **1)**	5/69 (7.3%)	5/58 (8.6%)	0/11	0.585
**Seems to be having less fun (** **≥** **1)**	23/70 (32.9%)	18/59 (30.5%)	5/11 (45.5%)	0.485
**Acts younger than children his or her age (** **≥** **1)**	16/68 (23.5%)	15/57 (26.3%)	1/11(9.1%)	0.437
**Does not listen to rules (** **≥** **1)**	28/68 (41.2%)	24/57 (42.1%)	4/11 (36.4%)	1.000
**Does not show feelings (** **≥** **1)**	16/69 (23.2%)	14/58 (24.1%)	2/11 (18.2%)	1.000
**Does not understand other people’s feeling (** **≥** **1)**	12/69 (17.4%)	10/58 (17.4%)	2/11 (18.2%)	1.000
**Teases others (** **≥** **1)**	27/67 (40.3%)	24/57 (42.1%)	3/10 (30%)	0.728
**Blames others for his or her troubles (** **≥** **1)**	21/67 (31.3%)	17/57 (29.8%)	4/10 (40%)	0.713
**Takes things that do not belong to him or her (** **≥** **1)**	9/67 (13.4%)	7/57 (12.3%)	2/10 (20%)	0.614
**Refuses to share (** **≥** **1)**	16/66 (24.4%)	13/56 (23.2%)	3/10 (30%)	0.695

* Fisher exact test for categorical variables, *t*-test for quantitative variables.

## Data Availability

The data presented in this study are available on request from the corresponding authors. The data are not publicly available due to privacy reasons.

## Supplementary Materials

The following supporting information can be downloaded at: https://www.mdpi.com/article/10.3390/children9091370/s1.
