# Supplementary material for: Perceived Psychological Impact on Children and Parents of Experiencing COVID-19 Infection in One or More Family Members
Source: Children (Basel). 2022 Sep 10;9(9):1370. doi: 10.3390/children9091370 (PMC9498072; doi:10.3390/children9091370)
Supplement: Supplementary file 1 [file children-09-01370-s001.zip › children-1845938-supplementary.pdf]

**Supplementary Material.**

English version of the web-based survey on-line distributed to 88 family clusters of COVID-19, enrolled at CovFC. The online survey was developed using the REDCap® platform (Vanderbilt University, Tennessee) hosted on the server of the University of Padova and it was shared by email to all parents that explicitly agreed to take part in the study, for the period of time from March 2020 to April 2021. The original survey was elaborated and distributed in Italian language.

**Dear Parents,**
**we kindly ask you to answer to some questions to help us understanding your family experience of SARS-CoV-2 related disease and isolation, from the psychological, relational and social points of view.**

Family code…………………..

Patient code…………………

Mother ☐ Father ☐ Legal tutor ☐ Other (specify ……………………………….)

date of survey compilation ….. /….. /……….

Education:

- Primary school degree
- Secondary school degree
- High school degree
- Bachelor degree

**Communication of and sharing the diagnosis**

1. In one word, how did you feel when you received your COVID – 19 diagnose?
2. Please indicate ONE IMAGE that may represent your emotional state when you received your diagnosis.
3. Other than the people you had direct contact with, did you inform anyone else you were positive for Covid – 19? yes ☐ no ☐
4. Did you feal that other people would treat you or your family differently when knowing of your diagnosis? yes ☐ no
5. Did anyone indeed act differently towards you or your family?
   yes ☐ no ☐
   If yes, then how was this difference? ☐ Positive ☐ Negative ☐ Both

**Familiar relationships**

Describe the composition of your cohabiting family before the diagnosis.

______________________________________________________________

Did the composition of your cohabiting family change after the diagnosis? (We ask you to specify any moving of people, separations, change in the living size for the family, due to recovery and/or isolation.)

yes ☐ no ☐
If yes, what changed? ______________________________________________________________________

Did your family format go back to the way it was before? yes ☐ no ☐

In your opinion, was your house adequate (in terms of space) to manage the isolation? yes ☐ no ☐

**Now think of the QUALITY of the family relationships within all members. We ask you to sign between 0 and 5 (Likert scale), where 0 means terrible relation, 5 means great relation:**

**Before the diagnosis:**
with the spouse/partner: 0__1 __2 __3 __4 __5 __
with the children: 0__1 __2 __3 __4 __5 __
among siblings: 0__1 __2 __3 __4 __5 __

**During COVID-19 related isolation:**
with the spouse/partner: 0__1 __2 __3 __4 __5 __
with the children: 0__1 __2 __3 __4 __5 __
among siblings: 0__1 __2 __3 __4 __5 __

**At the time of survey compilation (today):**
with the spouse/partner: 0__1 __2 __3 __4 __5 __
with the children: 0__1 __2 __3 __4 __5 __
among siblings: 0__1 __2 __3 __4 __5 __

**Before the diagnosis, were you working?**
Yes ☐ No ☐
Yes, as a:
☐ employee
☐ autonomous/entrepreneur
☐ permanent job, irregular
☐ occasional job, irregular
☐ others (specify) ______________________

**After the diagnosis and at the end of isolation, was there any change in your working schedule/time?**
Yes ☐ No ☐

If your working time has changed, please choose from the following:
☐ I have stayed home during isolation time and then I went back to work normally.
☐ I have done *smart working*
☐ I have reduced my working time
☐ I have lost my job
☐ other relevant changes (specify) __________________________________________

**Did the Covid -19 diagnosis have either positive or negative outcomes in your working life?**
Yes ☐ No ☐

☐ Positive outcome
☐ Negative outcome

**QUESTIONS INVESTIGATING THE CHILDREN/ADOLESCENT'S ADAPTATION**

**Please answer the following questions for each of your children.**

Child's date of birth. _________________

Positive to Covid - 19 yes □ no □ hospitalization yes □ no □

**Before the first Covid-19 epidemic, did you child have any developmental difficulty/disturb?** yes □ no □
(Here falls language and psychomotor development difficulties, emotional, relational, behavioural difficulties). If yes, specify ……………………………….

If yes, did the child pursue any rehabilitating, educational or therapeutical path?
yes □ no □
if yes, specify ……………………………………………

<u>**FOR EACH CHILD**</u>

**How do you think the adaptation of your child to the isolation time was?**

We ask you to sign between 0 and 5 (Likert scale), where 0 means very easy adaptation, 5 means very difficult adaptation.

**Did your child do distant learning?**
Yes □ no □

**Which electronic devices were used?**
- Pc
- Tablet
- Smart phone

**Did you have wi-fi connection?**
yes □ no □

**Did the family diagnosis and the isolation, in your opinion, determine changes in your child's adaptation?**
yes □ no □

<u>**ONLY FOR CHILDREN AGED 0-5**</u>

**Did the diagnosis of COVID-19 and the isolation affect the quality of the following aspects?** If yes, we ask you to explain how.

- Physical contact and physical self-care: yes □ no □ how?_ negative_ positive
- Breastfeeding/feeding: yes □ no □ how?_ negative_ positive
- Sleep/waking patterns patterns: yes □ no □ how?_ negative_ positive
- Bowel movements: yes □ no □ how?_ negative_ positive
- Language development quality: yes □ no □ how?_ negative_ positive
- Movement quality/quantity: yes □ no □ how?_ negative_ positive
- Playing quality: yes □ no □ how?_ negative_ positive
- Expressing and managing of emotions: yes □ no □ how?_ negative_ positive
- Quality of interactions with parents: yes □ no □ how?_ negative_ positive

- Quality of interactions with siblings: yes ☐ no ☐ how?_ negative_ positive

**ONLY FOR CHILDREN/ADOLESCENTS AGED 6-17**

| | | | |
|---|---|---|---|
| 1. Complains of aches/pains | never ☐ | sometimes ☐ | often ☐ |
| 2. Spends more time alone | never ☐ | sometimes ☐ | often ☐ |
| 3. Tires easily, has little energy | never ☐ | sometimes ☐ | often ☐ |
| 4. Fidgety, unable to sit still | never ☐ | sometimes ☐ | often ☐ |
| 5. Has trouble with a teacher | never ☐ | sometimes ☐ | often ☐ |
| 6. Less interested in school | never ☐ | sometimes ☐ | often ☐ |
| 7. Acts as if driven by a motor | never ☐ | sometimes ☐ | often ☐ |
| 8. Daydreams too much | never ☐ | sometimes ☐ | often ☐ |
| 9. Distracted easily | never ☐ | sometimes ☐ | often ☐ |
| 10. Is afraid of new situations | never ☐ | sometimes ☐ | often ☐ |
| 11. Feels sad, unhappy | never ☐ | sometimes ☐ | often ☐ |
| 12. Is irritable, angry | never ☐ | sometimes ☐ | often ☐ |
| 13. Feels hopeless | never ☐ | sometimes ☐ | often ☐ |
| 14. Has trouble concentrating | never ☐ | sometimes ☐ | often ☐ |
| 15. Less interest in friends | never ☐ | sometimes ☐ | often ☐ |
| 16. Fights with others | never ☐ | sometimes ☐ | often ☐ |
| 17. Absent from school | never ☐ | sometimes ☐ | often ☐ |
| 18. School grades dropping | never ☐ | sometimes ☐ | often ☐ |
| 19. Is down on him or herself | never ☐ | sometimes ☐ | often ☐ |
| 20. Visits doctor with doctor finding nothing wrong | never ☐ | sometimes ☐ | often ☐ |
| 21. Has trouble sleeping | never ☐ | sometimes ☐ | often ☐ |
| 22. Worries a lot | never ☐ | sometimes ☐ | often ☐ |
| 23. Wants to be with you more than before | never ☐ | sometimes ☐ | often ☐ |
| 24. Feels he or she is bad | never ☐ | sometimes ☐ | often ☐ |
| 25. Takes unnecessary risks | never ☐ | sometimes ☐ | often ☐ |
| 26. Gets hurt frequently | never ☐ | sometimes ☐ | often ☐ |
| 27. Seems to be having less fun | never ☐ | sometimes ☐ | often ☐ |
| 28. Acts younger than children his or her age | never ☐ | sometimes ☐ | often ☐ |
| 29. Does not listen to rules | never ☐ | sometimes ☐ | often ☐ |
| 30. Does not show feelings | never ☐ | sometimes ☐ | often ☐ |
| 31. Does not understand other people’s feelings | never ☐ | sometimes ☐ | often ☐ |
| 32. Teases others | never ☐ | sometimes ☐ | often ☐ |
| 33. Blames others for his or her troubles | never ☐ | sometimes ☐ | often ☐ |
| 34. Takes things that do not belong to him or her | never ☐ | sometimes ☐ | often ☐ |
| 35. Refuses to share | never ☐ | sometimes ☐ | often ☐ |

Note.

The “**Attention Problems” subscale** was derived from the sum of the following items: “Fidgety, unable to sit still”(item 4), "Daydreams too much”(item 8), "Distracted easily” (item 9), "Has trouble concentrating” (item 14), "Acts if is driven by a motor” (item 7). A cut-off > 7 indicates an impairment condition.

The **“Internalizing problems” subscale** was derived from the sum score of the following items: “Feels sad or unhappy” (item 11), “Feels hopeless” (item 13), “Is down on him or herself” (item 19), “Worries a lot” (item 22). A cut-off > 5 indicates an impairment condition.

The **“Externalizing problems” subscale** was derived from the sum of the following items: “fight with others” (item 16), “does not listen to rules” (item 29), “does not understand other people feelings” (item 31), “teases others” (item 32), “blamed others for his / her troubles” (item 33), “takes things that do not belong to him/her” (item 34), “refuses to share” (item 35). A cut-off > 7 indicates an impairment condition.
